# Amino acids and risk of colon adenocarcinoma: a Mendelian randomization study

**DOI:** 10.1186/s12885-023-11514-w

**Published:** 2023-10-28

**Authors:** Yuanyuan Wang, Zhihan Jia, Qingjun Wang, Zhitu Zhu

**Affiliations:** 1 Cancer Clinical Research Ward, The First Affiliated Hospital of Jinzhou Medical University, Jinzhou, 121000 China; 2 Department of Oncology, The First Affiliated Hospital of Jinzhou Medical University, Jinzhou, 121000 China; 3 Liaoning Provincial Key Laboratory of Clinical Oncology Metabonomics, Institute of Clinical Bioinformatics, Cancer Center of Jinzhou Medical University, The First Affiliated Hospital of Jinzhou Medical University, Jinzhou, 121000 China

**Keywords:** Alanine, Colon adenocarcinoma, Protective factors, Prognosis

## Abstract

**Background:**

The existence of amino acid metabolic reprogramming in tumor cells is well established. However, the potential correlation between blood amino acids and the risk of colon adenocarcinoma remains largely unexplored.

**Methods:**

We utilized Mendelian randomization (MR) analysis to examine the association between 20 amino acids in the blood and the risk of colon adenocarcinoma. Additionally, reverse MR analysis was employed to identify the presence of reverse causality. A two-step MR analysis was conducted to ascertain the potential mediating effect. Lastly, the alanine detection data from colon adenocarcinoma patients in our hospital were utilized to investigate the differences in alanine levels among healthy individuals and patients with colon cancer, as well as among patients with different stages and locations of colon cancer. Furthermore, a Kaplan–Meier curve was employed to examine the correlation between alanine and overall survival, followed by the implementation of COX univariate analysis.

**Results:**

The results of our study indicate that there is an inverse correlation between alanine and the risk of colon adenocarcinoma. Additionally, we found no significant evidence to support a causal relationship between colon adenocarcinoma and alanine. Furthermore, our analysis revealed that alanine aminotransferase (ALT) and blood glucose do not act as mediators in this causal pathway. Moreover, individuals diagnosed with colon adenocarcinoma exhibited a significant decrease in alanine levels, particularly in cases of stage IV colon adenocarcinoma with distant metastasis. Additionally, elevated alanine levels were associated with improved overall survival rates among colon adenocarcinoma patients.

**Conclusions:**

The results of this study indicate that alanine exhibits protective characteristics against the onset of colon adenocarcinoma and may play a role in promoting a more favorable disease prognosis. Consequently, dietary interventions aimed at increasing alanine intake may serve as a potential strategy for the prevention and treatment of colon adenocarcinoma.

**Supplementary Information:**

The online version contains supplementary material available at 10.1186/s12885-023-11514-w.

## Background

Colon cancer is a malignancy with a high incidence rate that poses a significant threat to human health [1]. It is imperative to identify the etiology and pathogenesis of colon cancer to facilitate its prevention and treatment. Recent research has demonstrated that tumors exhibit abnormal metabolism, and metabolic reprogramming plays a crucial role in tumor progression and response to immunotherapy [2, 3]. Amino acid metabolic reprogramming is a critical component of tumor metabolic reprogramming, which profoundly influences various biological behaviors of tumors [4, 5].

Previous research has demonstrated that the amino acid composition in the blood of individuals with gastric [6, 7] and breast [8] cancer is notably aberrant in comparison to healthy individuals, which can be utilized for the purpose of tumor identification and early detection. And, investigations have also revealed that the amino acid composition in the blood of patients with colon cancer is significantly anomalous [9, 10]. Several studies have suggested that the consumption of branched chain amino acids may increase the probability of mortality linked to colorectal cancer [11, 12]. The identification of specific amino acids that serve as risk factors for the development of colon cancer remains unknown. Additionally, the potential protective or cancer promoting effects of individual amino acids in relation to colon cancer are unclear. Conducting prospective amino acid interventions to observe their impact on colon cancer incidence is deemed unethical. Consequently, we employed computer based Mendelian randomization (MR) analysis to examine the causal relationship between blood amino acid levels and the risk of colon cancer.

The genetic epidemiological approach known as Mendelian randomization (MR) employs single nucleotide polymorphisms (SNPs) that exhibit strong associations with the exposure as instrumental variables (IVs) to estimate the potential causal relationship between the exposure and the outcome [13]. Since genotypes are presumed to be randomly allocated in the process of gamete formation, the introduction of the IV model largely resolves the issue of confounding in observational studies, particularly the bias effect of unmeasured confounders on causal inference [14]. This approach has been shown to be effective in addressing causal questions in various research.

Colon cancer encompasses a diverse range of pathological subtypes, including adenocarcinoma, mucinous adenocarcinoma, signet ring cell carcinoma, squamous cell carcinoma, adenosquamous carcinoma, and medullary carcinoma, among others. Adenocarcinoma represents the predominant pathological subtype [15]. The present study investigated the relationship between 20 amino acids in the blood and the risk of colon adenocarcinoma. The results revealed a negative association between alanine and the risk of colon cancer. Furthermore, the serum alanine levels of colon adenocarcinoma patients and normal individuals were analyzed, and a significant decrease in alanine content was observed in colon cancer patients. Notably, patients with high alanine content exhibited a favorable prognosis. These findings suggest that alanine may act as an inhibitory factor in the onset and progression of colon adenocarcinoma. The manipulation of alanine levels, including the supplementation of alanine in the diet, may serve as a viable strategy for preventing and treating colon adenocarcinoma.

## Materials and methods

### Study design

**Fig. 1:**
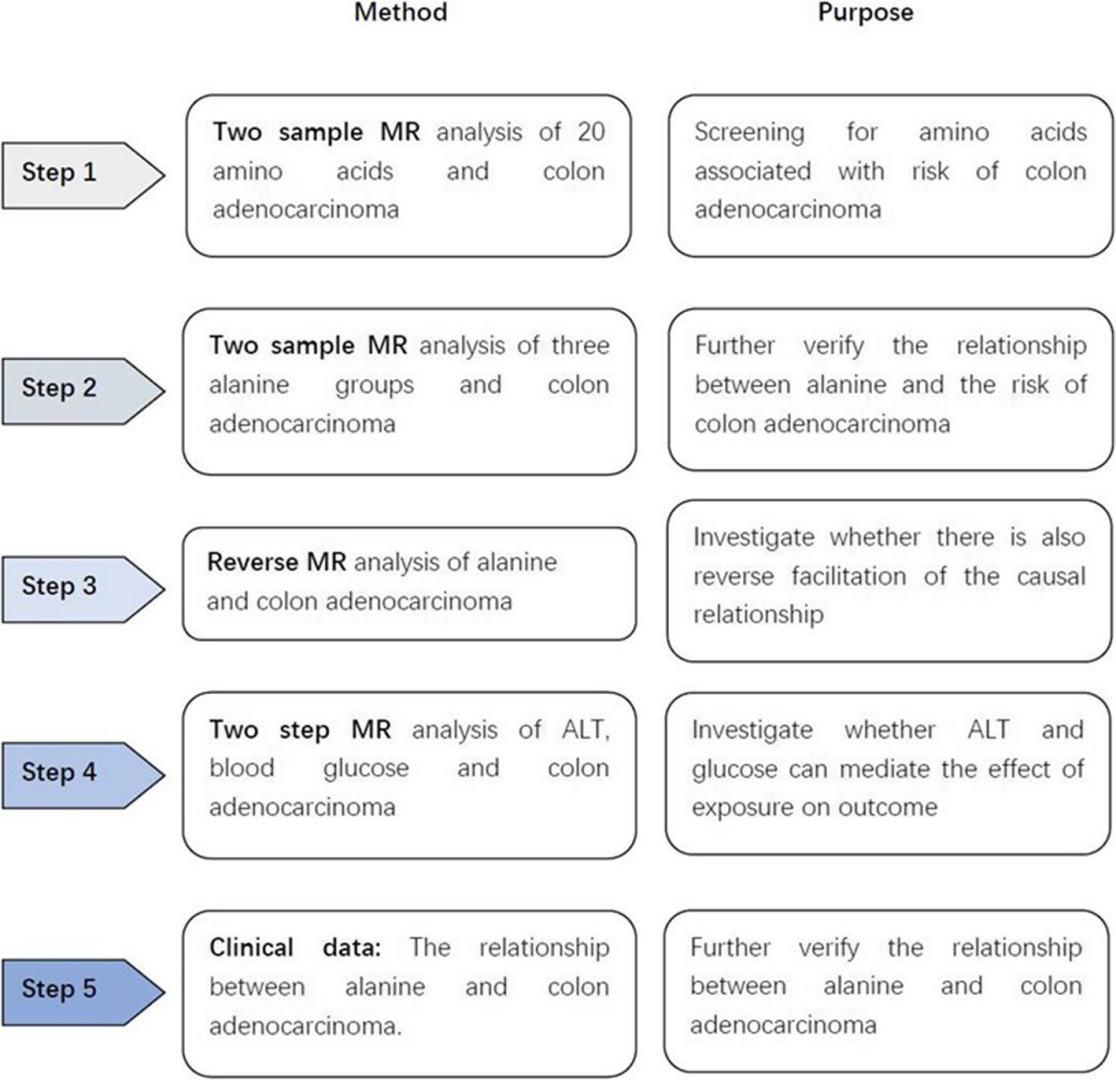
Study design

Initially, we employed a two sample Mendelian randomization approach to investigate the correlation between 20 amino acids and the risk of colon adenocarcinoma. Our findings revealed that alanine exhibited a significant association with the risk of colon adenocarcinoma. Subsequently, we conducted a reanalysis of the relationship between alanine and the risk of colon adenocarcinoma by utilizing three sets of alanine data while imposing the condition of linkage disequilibrium (LD). In order to examine the potential for reverse causality and mediating effects, we conducted a reverse analysis of the impact of colonic adenocarcinoma on alanine. Additionally, we investigated the possibility of ALT and gluconic serving as mediating factors in influencing the outcome. Finally, we utilized hospital data to observe the levels of alanine in the blood of patients diagnosed with colon adenocarcinoma and to explore the correlation between alanine levels and the stage and prognosis of the disease. A schematic of the study design is shown in Fig. 1.

### Data sources

The genome-wide association study (GWAS) datasets for 20 amino acids, colon adenocarcinoma, Alanine aminotransferase, and Fasting blood glucose were obtained from the IEU GWAS database (https://gwas.mrcieu.ac.uk/), which were limited to the European population. Blood amino acid data, along with relevant clinical pathological and prognostic information, were collected from 83 healthy individuals and 236 patients with colon adenocarcinoma at the First Affiliated Hospital of Jinzhou Medical University. After a minimum of 12 h of fasting, blood samples were collected from all participants. Our previously published article provides a comprehensive method for detecting amino acids [7]. Blood amino acid data was collected from healthy individuals aged 50–80 years in addition to patients diagnosed with colon adenocarcinoma within the same age range. The gender composition of both groups was found to be similar. This study was approved by the institutional ethics committee of The First Affiliated Hospital of Jinzhou Medical University (No.202347).

The identification code for colon adenocarcinoma is finn-b-c3_COLON_ADENO, while the identification code for Alanine aminotransferase is bbj-a-6, and for Fasting blood glucose is ebi-a-GCST90002232. Table 1 presents comprehensive information on all amino acids.

**Table 1:** Summary of the amino acid data sets

**Exposure**	**ID**	**Population**	**Sample size**	**Number of SNPs**	**Year**	**Author**
Alanine	met-d-Ala	European	115,074	12,321,875	2020	Borges CM
	met-c-840	European	24,796	12,091,566	2016	Kettunen
	met-a-469	European	7,788	2,545,552	2014	Shin
Arginine	met-a-347	European	7,528	2,545,579	2014	Shin
Asparagine	met-a	European	7,761	2,545,507	2014	Shin
Aspartate	met-a	European	7,721	2,545,425	2014	Shin
Aysteine	met-a	European	7,692	2,545,727	2014	Shin
Glutamine	met-d	European	114,750	12,321,875	2020	Borges CM
Glutamate	met-a	European	7,804	2,545,537	2014	Shin
Glycine	met-d	European	114,972	12,321,875	2020	Borges CM
Histidine	met-d	European	114,895	12,321,875	2020	Borges CM
Isoleucine	met-d	European	115,075	12,321,875	2020	Borges CM
Leucine	met-d	European	115,074	12,321,875	2020	Borges CM
Lysine	met-a	European	7,812	2,545,686	2014	Shin
Methionine	met-a	European	7,795	2,545,691	2014	Shin
Phenylalanine	met-d	European	115,025	12,321,875	2020	Borges CM
Proline	met-a	European	7,816	2,545,669	2014	Shin
Serine	met-a	European	7,796	2,545,555	2014	Shin
Threonine	met-a	European	6,020	2,545,896	2014	Shin
Tryptophan	met-a	European	7,804	2,545,641	2014	Shin
Tyrosine	met-d	European	114,911	12,321,875	2020	Borges CM
Valine	met-d	European	115,048	12,321,875	2020	Borges CM

### Selection of instrumental variables

In the present MR study, SNPs that exhibited a significant association with blood 20 amino acid at the genome-wide level of significance (P value < 5 × 10 – 8) and demonstrated no linkage disequilibrium (LD) with other SNPs (r2 < 0.001 within a clumping window of 10,000 kb) were employed as instruments for these blood amino acid. However, due to insufficient SNP availability for certain amino acids under the aforementioned conditions, the p value for arginine, proline, glutamic acid, and lysine was adjusted to 5e-7, while the p value for cysteine, threonine, and aspartic acid was set to 5e-6.

Upon conducting a screening, it was discovered that alanine exhibited an association with the risk of colon adenocarcinoma. To ensure accuracy, we utilized three sets of alanine exposure data from the IEU GWAS database and identified SNPs that demonstrated a significant association with blood alanine at the genome-wide level (P value < 5 × 10 – 8) and were not in linkage disequilibrium (LD) with other SNPs (r2 < 0.0001 within a clumping window of 10,000 kb) as instrumental variables for alanine. Due to a limited number of SNPs available for instrumental variable analysis in met-a-469, the p-value was adjusted to 5e-7.

Furthermore, a reverse Mendelian randomization analysis was conducted utilizing colon adenocarcinoma as the exposure and alanine as the outcome. SNPs as an instrumental variable associated with colon adenocarcinoma were established at the genome-wide significance level (P value < 5 × 10 – 6) and were required to exhibit no linkage disequilibrium (LD) with other SNPs (r2 < 0.01 within a clumping window of 10,000 kb).

### Statistical analysis

The Inverse Variance Weighted (IVW) method was utilized as the primary approach for our MR analysis to investigate the causal impact of 20 blood amino acid levels on the risk of colon adenocarcinoma. Additionally, we employed other methods such as Weighted Median and Weighted Mode. Subsequently, a series of sensitivity analyses were conducted, including Heterogeneity (Cochran's Q statistics), Horizontal Pleiotropy (MR-Egger intercept analysis), and Leave-One-Out analysis. We also performed an MR-PRESSO analysis and utilized the MR-PRESSO outlier test method to remove outlier SNPs.

The present study conducted MR analysis, difference analysis of alanine level in blood among normal individuals and those diagnosed with colon adenocarcinoma, difference analysis of alanine level across various stages, Kaplan–Meier method was used to draw the survival curve, and the Log-rank test was used for comparison, with P < 0.05 as statistically significant. and univariate Cox analysis to examine the relationship between age, gender, stage, differentiation, and prognosis. All of the aforementioned analyses were deemed statistically significant at a p value of less than 0.05. Kaplan–Meier method was used to draw the survival curve, and the Log-rank test was used for comparison, with P < 0.05 as statistically significant.

## Results

### Two-sample Mendelian randomization analysis of 20 amino acids and colon adenocarcinoma

Utilizing the IVW method, an MR analysis was conducted to investigate the correlation between 20 blood amino acids and the risk of colon adenocarcinoma. The results revealed a causal association between alanine and colonic adenocarcinoma, with a significance level of p < 0.05. Conversely, no significant relationship was observed between other amino acids and the risk of colon adenocarcinoma, with a p value exceeding 0.05. The detailed results are in Table 2.

**Table 2:** MR analysis for 20 amino acids in blood associations with colon adenocarcinoma risk

**Exposure**	**ID**	**No. of SNPs**	**OR**	**95% CI**	***P* value**
**Alanine**	met-d-Ala	32	0.66	(0.45-0.98)	**0.042**
**Arginine**	met-a-347	2	1.60	(0.02-111.05)	0.827
**Asparagine**	met-a-638	2	1.94	(0.12-9.34)	0.639
**Aspartate**	met-a-388	3	1.07	(0.12-31.03)	0.951
**Aysteine**	met-a-455	11	1.48	(0.46-4.74)	0.512
**Glutamine**	met-d-Gin	40	1.17	(0.87-1.57)	0.290
**Glutamate**	met-a-466	2	11.74	(0.56-243.96)	0.112
**Glycine**	met-d-Gly	43	1.03	(0.89-1.19)	0.699
**Histidine**	met-d-His	15	1.04	(0.57-1.90)	0.888
**Isoleucine**	met-d-Ile	10	0.85	(0.30-2.43)	0.767
**Leucine**	met-d-Leu	16	1.01	(0.53-1.96)	0.968
**Lysine**	met-a-326	2	0.20	(0.00-30.86)	0.530
**Methionine**	met-a-327	2	7.94	(0.00-19241.33)	0.602
**Phenylalanine**	met-d-Phe	8	1.31	(0.77-2.21)	0.320
**Proline**	met-a-355	4	2.02	(0.25-16.49)	0.512
**Serine**	met-a-464	3	1.66	(0.14-18.99)	0.684
**Threonine**	met-a-324	13	0.98	(0.10-9.54)	0.983
**Tryptophan**	met-a-304	18	0.06	(0.00-2.32)	0.130
**Tyrosine**	met-d-Tyr	31	0.99	(0.72-1.36)	0.956
**Valine**	met-d-Val	19	0.95	(0.55-1.63)	0.841

### Two-sample Mendelian randomization analysis of alanine and colon adenocarcinoma

**Fig. 2:**
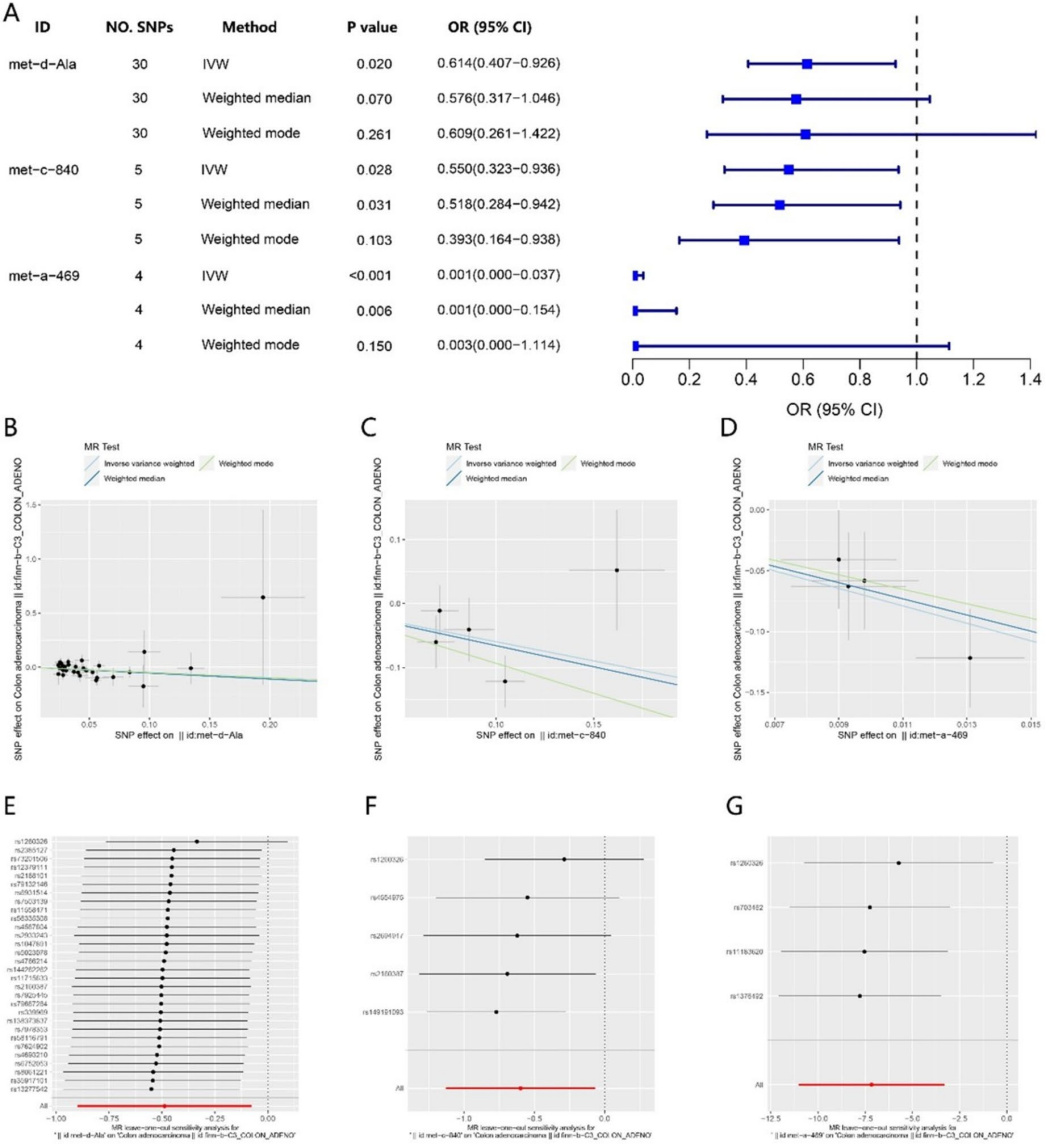
Two-sample MR analysis of alanine and risk of colon adenocarcinoma. **A** Forest plots representing the MR estimates and 95% CI values of the causal effects of Ala. **B**-**D** Gene prediction of the association between Ala levels and risk of colon cancer based on different MR methods from 3 sets of alanine data analysis. **E**–**G** Leave-one-out analysis from 3 sets of alanine data

To ascertain the precise association between alanine and the risk of colon adenocarcinoma, we employed three MR techniques to scrutinize the correlation between three distinct alanine exposure datasets and the risk of colon adenocarcinoma. Our analysis of alanine with an ID of met-d-Ala and the risk of colon adenocarcinoma revealed that the IVW method yielded a p value of less than 0.05, the Weighted median method yielded a p value close to 0.05, and the OR value was less than 1 (Fig. 2A). Further examination of the alanine exposure data from two additional groups, identified as met-c-840 and met- a-469, demonstrated that both the IVW and Weighted median methods produced p values below 0.05, and the OR value remained less than 1 (Fig. 2A). The examination of three sets of alanine exposure data revealed a negative correlation between alanine and the likelihood of colon adenocarcinoma (Fig. 2B-D). Additionally, the leave-one-out analysis indicated that a single SNP did not impact the results (Fig. 2E-G). Other sensitivity analyses also revealed that the MR-Egger Intercept p value and the Cochran Q test p value were both greater than 0.05. Detailed results can be found in supporting materials met-d-Ala, met-c-840, and met-a-469.

### Reverse Mendelian randomization analysis of alanine and colon adenocarcinoma

**Fig. 3:**
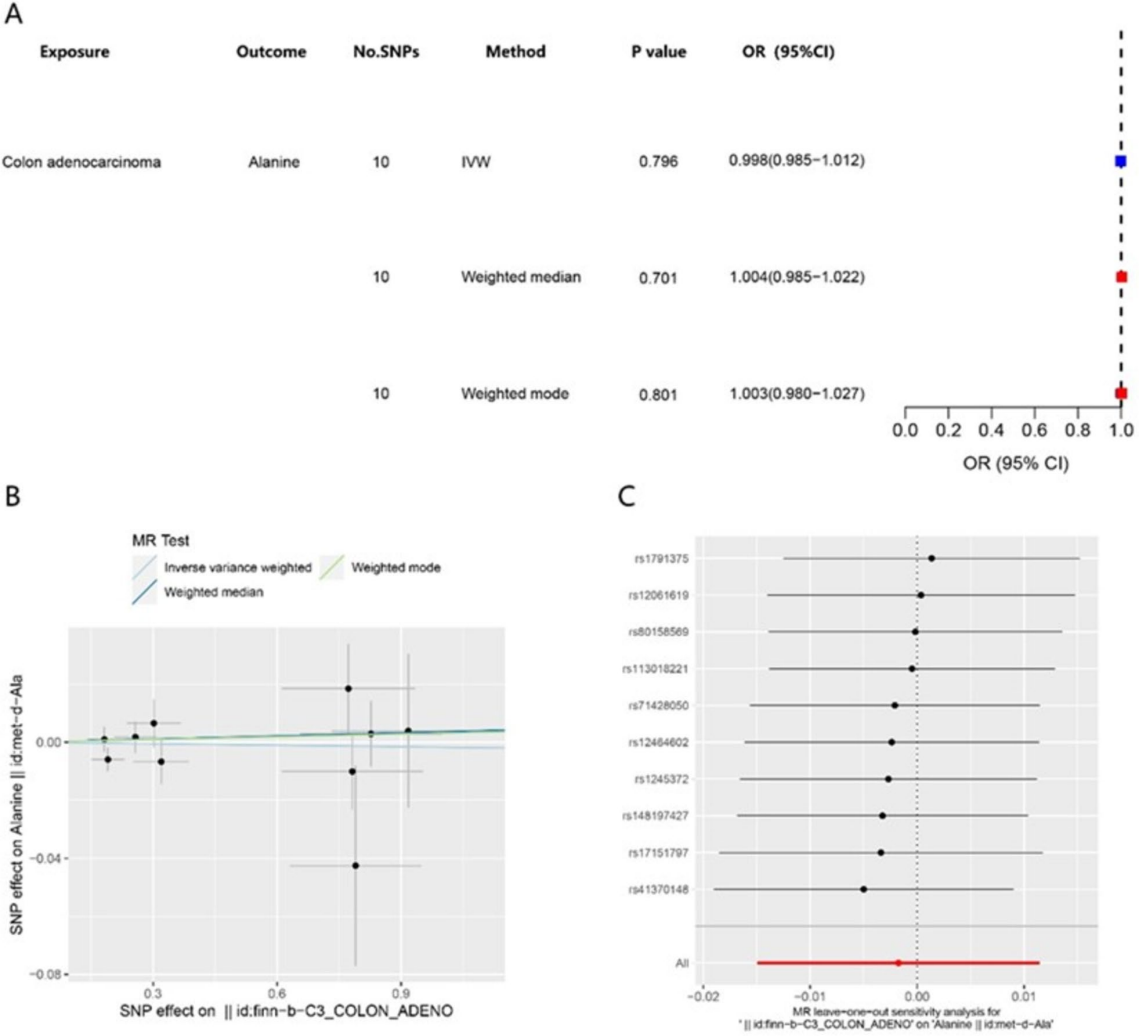
Reverse Mendelian randomization analysis of alanine and colon adenocarcinoma. **A** Forest plots representing the MR estimates and 95% CI values of the causal effects of colon adenocarcinoma. **B** Gene prediction of the association between colon adenocarcinoma levels and risk of Ala based on different MR methods. **C** Leave-one-out analysis

To investigate the potential reverse causal association between alanine and colon adenocarcinoma, a Mendelian randomization (MR) analysis was conducted with colon adenocarcinoma as the exposure variable and alanine as the outcome variable. The results of the analysis revealed a nonsignificant relationship between the two variables, with a p value greater than 0.05 (Fig. 3A). There is no obvious correlation between them (Fig. 3B). The Leave-one-out analysis also demonstrated that the relationship was not sensitive (Fig. 3C).

### Two-step MR analysis of the mediating effect of ALT and Fasting blood glucose

No significant causal relationship was observed between alanine and ALT, Fasting blood glucose (FBG), as well as between ALT, Fasting blood glucose, and colon adenocarcinoma, as indicated by P values greater than 0.05 (Table 3).

**Table 3:** Two-step MR analysis of the mediating effect of ALT and Fasting blood glucose

Exposure	Outcome	No. of SNPs	OR (95% CI)	*P *value	Exposure	Outcome	No. of SNPs	OR (95% CI)	*P *value
**Alanine**	ALT	24	0.96(0.87-1.07)	0.47	ALT	COAD	22	0.83(0.43-1.59)	0.58
**Alanine**	FBG	32	1.08(0.97-1.19)	0.16	FBG	COAD	63	1.10(0.68-1.78)	0.71

### Alanine levels in the blood of patients with colon adenocarcinoma and its relationship with stage and prognosis

**Fig. 4:**
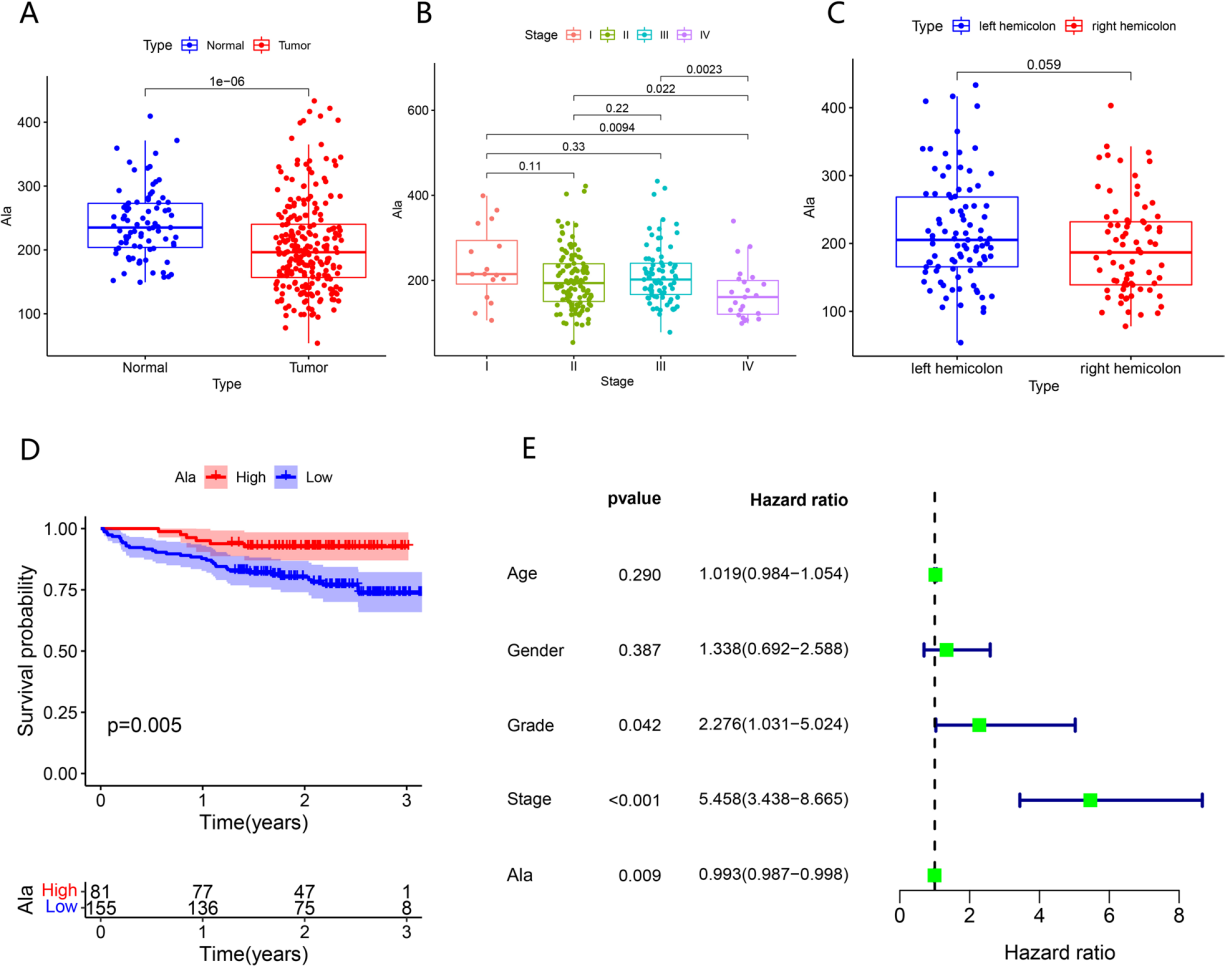
Alanine levels in the blood of patients with colon adenocarcinoma and its relationship with stage and prognosis. **A** Difference of Alanine Level in Blood between Normal People and Patients with Colon adenocarcinoma. **B** Relationship between alanine level and stage in patients with colon adenocarcinoma. **C** Relationship between alanine level and OS in patients with colon adenocarcinoma. **D** Univariate COX analysis was used to analyze the relationship between age, sex, differentiation, stage, ala, and OS of colon adenocarcinoma

Through our data analysis, it was determined that the blood alanine levels of individuals diagnosed with colon adenocarcinoma were significantly lower in comparison to those of healthy individuals (Fig. 4A). This difference was found to be statistically significant (Fig. 4A). Furthermore, a significant decrease in alanine content was observed in patients with stage IV colon adenocarcinoma in comparison to those without metastasis (Fig. 4B). This difference was also found to be statistically significant (Fig. 4B). In addition, a comparison was conducted between the levels of alanine in patients diagnosed with left hemicolon and right hemicolon adenocarcinomas. The findings revealed that the levels of alanine in right hemicolon adenocarcinomas were comparatively lower than those observed in left hemicolon adenocarcinomas. However, it is worth noting that this disparity did not reach statistical significance (P = 0.059) (Fig. 4C). Prognostic analysis revealed that patients with elevated alanine levels exhibited a favorable prognosis (Fig. 4D). Additionally, univariate COX analysis indicated that alanine served as a positive prognostic factor for colon adenocarcinoma (Fig. 4E).

## Discussion

Amino acids serve as substrates for protein synthesis and are integral to the proliferation and progression of tumors. Moreover, amino acids comprise a vast and intricate metabolic network that is closely interconnected with other metabolic pathways [4]. It is not known whether amino acids in the blood are a risk factor for colon adenocarcinoma. Genetically predicted circulating alanine and ALT levels have been associated with altered prostate cancer risk in an MR study [16].

Mendelian randomization is a new epidemiological analysis method, which can reduce the bias of conventional epidemiological analysis methods and the influence of confounding factors [13]. Given the ethical concerns surrounding prospective intervention studies, we also used MR methods to analyze the causal relationship between 20 amino acids and colon adenocarcinoma. We found that alanine was inversely associated with the risk of colon adenocarcinoma.

Subsequently, in order to accurately determine the relationship between alanine and the risk of colon adenocarcinoma, we conducted an analysis of the relationship between alanine and colon adenocarcinoma risk across three alanine groups. The resulting p values of IVW were all less than 0.05. Sensitivity analysis revealed that the MR-Egger Intercept p value and the Cochran Q test P value were both greater than 0.05. Leave-one-out analysis also found that the results were not affected by a single SNP. All of these suggest that alanine is negatively associated with the risk of colon adenocarcinoma and that the results are reliable.

Our previous study also found that the content of alanine in the blood of colon cancer patients was significantly lower than that of colon polyps, and the difference was statistically significant [10]. Furthermore, in order to ascertain whether a reverse causality exists between alanine and colon adenocarcinoma, a two-sample MR analysis was conducted with colon adenocarcinoma serving as the exposure factor and alanine as the outcome. The findings indicated that there is no reverse causal relationship between alanine and colon adenocarcinoma.

Alanine is a crucial nonessential amino acid that serves a significant function in the maintenance of blood glucose stability [17], cellular protection against oxidative damage [18], and collagen synthesis [19], among other roles. The primary metabolic pathway for alanine in the body is through alanine aminotransferase (ALT), which converts it to pyruvate. Pyruvate serves as the end product of glycolysis and the initial substrate for the tricarboxylic acid (TCA) cycle. Pyruvate facilitates the ability of tumor cells to adapt to deleterious conditions within the tumor microenvironment by reconfiguring metabolic pathways [20]. The progression of breast cancer metastasis is contingent upon pyruvate-mediated collagen-based extracellular matrix remodeling [21].

Consequently, we conducted an analysis to determine if glucose and ALT acted as mediating factors in the progression of colon adenocarcinoma via alanine. The findings indicated that the impact of alanine on glucose and ALT did not demonstrate statistical significance.

So, alanine may serve as a protective factor against colon adenocarcinoma by inhibiting its occurrence. Our research findings indicate a significant decrease in blood alanine levels among colon adenocarcinoma patients compared to healthy individuals. Additionally, studies have demonstrated that the reduction of alanine can intensify the nonadherent growth of colon cancer HCT116 cells, which is a characteristic feature of aggressive tumors [22]. Furthermore, alanine plays a role in collagen synthesis [19, 21], which is closely linked to tumor invasion and metastasis [23, 24].

Simultaneously, our investigation revealed a notable decrease in alanine levels among patients diagnosed with stage IV colon adenocarcinoma with metastasis in comparison to those without metastasis. Our prognostic assessment demonstrated that patients exhibiting elevated alanine levels exhibited a more favorable prognosis, indicating that alanine elevation serves as a promising prognostic indicator for colon adenocarcinoma. The underlying mechanism may be attributed to its impact on invasion and metastasis.

## Conclusions

It is widely acknowledged that tumors exhibit aberrant amino acid metabolism, resulting in abnormal amino acid metabolites in the bloodstream of cancer patients. However, the specific amino acids in plasma that serve as risk or protective factors for cancer have yet to be definitively identified. The findings of this study demonstrate an inverse relationship between alanine and the risk of colon adenocarcinoma, as well as a correlation between alanine levels and the prognosis of this malignancy, indicating that alanine may serve as a protective factor against colon adenocarcinoma. The discovery holds immense potential significance as elevating alanine levels may be a viable strategy for the prevention and treatment of colon cancer in the future.

### Supplementary Information

**Additional file 1.** The following supporting information can be downloaded at: www.mdpi.com/xxx/s1, supporting materials met-d-Ala; supporting materials met-a-469; supporting materials met-c-840; supporting materials reverse MR. Patient clinical pathological information, alanine data, and survival information can be downloaded from supplementary materials

## Data Availability

This published article and its Supplementary Materials contain all data that were generated or analyzed during the course of this study. The codes that were generated or utilized during the study can be obtained from the corresponding author upon request.

## Abbreviations

MR: Mendelian randomization
ALT: Alanine aminotransferase
SNPs: Single nucleotide polymorphisms
LD: Linkage disequilibrium
GWAS: Genome-wide association study
IVW: Inverse Variance Weighted
FBG: Fasting blood glucose
TCA: Tricarboxylic acid

## References

[1] Bray F, Ferlay J, Soerjomataram I, Siegel RL, Torre LA, Jemal A (2018). Global cancer statistics 2018: GLOBOCAN estimates of incidence and mortality worldwide for 36 cancers in 185 countries. CA Cancer J Clin.

[2] Faubert B, Solmonson A, DeBerardinis RJ. Metabolic reprogramming and cancer progression. Science. 2020;368(6487). 10.1126/science.aaw5473. 10.1126/science.aaw5473 PMC7227780 32273439

[3] Xia L, Oyang L, Lin J, Tan S, Han Y, Wu N (2021). The cancer metabolic reprogramming and immune response. Mol Cancer.

[4] Lieu EL, Nguyen T, Rhyne S, Kim J (2020). Amino acids in cancer. Exp Mol Med.

[5] Peng H, Wang Y, Luo W (2020). Multifaceted role of branched-chain amino acid metabolism in cancer. Oncogene.

[6] Jing F, Hu X, Cao Y, Xu M, Wang Y, Jing Y (2018). Discriminating gastric cancer and gastric ulcer using human plasma amino acid metabolic profile. IUBMB Life.

[7] Shi LY, Wang YY, Jing Y, Xu MH, Zhu ZT, Wang QJ (2021). Abnormal arginine metabolism is associated with prognosis in patients of gastric cancer. Transl Cancer Res..

[8] Hu L, Gao Y, Cao Y, Zhang Y, Xu M, Wang Y (2016). Identification of arginine and its "Downstream" molecules as potential markers of breast cancer. IUBMB Life.

[9] Barberini L, Restivo A, Noto A, Deidda S, Fattuoni C, Fanos V (2019). A gas chromatography-mass spectrometry (GC-MS) metabolomic approach in human colorectal cancer (CRC): the emerging role of monosaccharides and amino acids. Ann Transl Med..

[10] Jing Y, Wu X, Gao P, Fang Z, Wu J, Wang Q (2017). Rapid differentiating colorectal cancer and colorectal polyp using dried blood spot mass spectrometry metabolomic approach. IUBMB Life.

[11] Katagiri R, Song M, Zhang X, Lee DH, Tabung FK, Fuchs CS (2020). Dietary intake of branched-chain amino acids and risk of colorectal cancer. Cancer Prev Res (Phila).

[12] Long L, Yang W, Liu L, Tobias DK, Katagiri R, Wu K (2020). Dietary intake of branched-chain amino acids and survival after colorectal cancer diagnosis. Int J Cancer.

[13] Kajantie E, Osmond C, Barker DJ, Forsén T, Phillips DI, Eriksson JG (2005). Size at birth as a predictor of mortality in adulthood: a follow-up of 350 000 person-years. Int J Epidemiol.

[14] Davies NM, Holmes MV, Davey SG (2018). Reading Mendelian randomisation studies: a guide, glossary, and checklist for clinicians. BMJ.

[15] Treanor D, Quirke P (2007). Pathology of colorectal cancer. Clin Oncol (R Coll Radiol).

[16] Yang S, Song J, Yang H, Liu W, Jiang Y, Sun X (2022). Genetically predicted circulating concentrations of alanine and alanine aminotransferase were associated with prostate cancer risk. Clin Epidemiol.

[17] Jubouri M, Talarico G, Weber JM, Mennigen JA. Alanine alters the carbohydrate metabolism of rainbow trout: glucose flux and cell signaling. J Exp Biol. 2021;224(15). 10.1242/jeb.232918. 10.1242/jeb.232918 34374410

[18] Petersen KF, Dufour S, Cline GW, Shulman GI (2019). Regulation of hepatic mitochondrial oxidation by glucose-alanine cycling during starvation in humans. J Clin Invest.

[19] Röder K (2022). The effects of glycine to alanine mutations on the structure of GPO collagen model peptides. Phys Chem Chem Phys.

[20] Prochownik EV, Wang H. The Metabolic Fates of Pyruvate in Normal and Neoplastic Cells. Cells. 2021;10(4). 10.3390/cells10040762. 10.3390/cells10040762 PMC8066905 33808495

[21] Elia I, Rossi M, Stegen S, Broekaert D, Doglioni G, van Gorsel M (2019). Breast cancer cells rely on environmental pyruvate to shape the metastatic niche. Nature.

[22] Muthusamy T, Cordes T, Handzlik MK, You L, Lim EW, Gengatharan J (2020). Serine restriction alters sphingolipid diversity to constrain tumour growth. Nature.

[23] Chen D, Liu Z, Liu W, Fu M, Jiang W, Xu S (2021). Predicting postoperative peritoneal metastasis in gastric cancer with serosal invasion using a collagen nomogram. Nat Commun.

[24] Wishart AL, Conner SJ, Guarin JR, Fatherree JP, Peng Y, McGinn RA, et al. Decellularized extracellular matrix scaffolds identify full-length collagen VI as a driver of breast cancer cell invasion in obesity and metastasis. Sci Adv. 2020;6(43). 10.1126/sciadv.abc3175. 10.1126/sciadv.abc3175 PMC7577726 33087348

